# A Computational Model of Working Memory Integrating Time-Based Decay and Interference

**DOI:** 10.3389/fpsyg.2018.00416

**Published:** 2018-04-03

**Authors:** Benoît Lemaire, Sophie Portrat

**Affiliations:** CNRS, LPNC UMR 5105, Université Grenoble Alpes, Grenoble, France

**Keywords:** working memory, time-based decay, interference, computational modeling, simulations, refreshing, long-term memory

## Abstract

There is still a strong debate in the working memory literature about the cause of forgetting, with many articles providing evidence for the existence of temporal decay and as many publications providing evidence compatible with interference being the only mechanism involved in forgetting. In order to reconcile the two views, this article describes TBRS^∗^-I (for Time-Based Resource-Sharing^∗^-Interference), a computational model of working memory which incorporates an interference-based mechanism to the decay-based implementation TBRS^∗^ within the TBRS theoretical framework. At encoding, memoranda are associated to their context, namely their position in the list. Temporal decay decreases the strength of these associations, but a refreshing process may reactivate it during free time. Distractors may alter the distributed representation of memoranda but refreshing can restore them based on the long-term memory representations. Refreshing is therefore twofold: reactivation plus restoration, each one counteracting the detrimental time-based and interference-based decays, respectively. Two types of interference are implemented: interference by confusion which depends on the degree of overlap between memoranda and distractors and interference by superposition which depends on the similarity between them. TBRS^∗^-I was tested on six benchmark findings on retention-interval and distractor-processing effects by means of millions of simulations testing the effects of seven factors on memory performance: the number of memoranda, the duration of distractor attentional capture, the duration of free time, the number of distractors, the amount of overlap between memoranda and distractors, the similarity between memoranda and distractors and the homogeneity of distractors (all identical or all distinct). TBRS^∗^-I replicated classical effects and proved to be a suitable hybrid model integrating both interference and time-based decay. The article also discusses the compatibility of TBRS^∗^-I with a unitary or dual view of memory and the issue of integrating time and interference in a single model. Computer codes and data are available at https://osf.io/65sna/.

## Introduction

Working memory is a cognitive system that allows people to maintain information for short periods of time, in order to accomplish all sorts of cognitive activities. This memory system is able to store and maintain information even if other information has to be processed at the same time. However, some memoranda may still be forgotten according to the characteristics of the situation. Therefore, cognitive psychologists have investigated for decades the reasons for this loss of information. There is an old and strong debate between decay and interference explanations, that is between a time-based passive damaging of memory traces and an active distortion of these traces by intervening events (McGeoch, 1932). However, the debate is more about the existence or not of temporal decay, because proponents of temporal decay do not deny the existence of interference whereas defenders of interference often claim that time-based decay is a red herring.

In the domain of working memory, this opposition turned out to be quite strong in the past years with numerous articles proving the existence of temporal decay (Portrat et al., 2008; Barrouillet and Camos, 2009; Barrouillet et al., 2011b), to which studies against decay replied (Lewandowsky and Oberauer, 2009, 2015; Lewandowsky et al., 2010; Oberauer and Lewandowsky, 2013, 2014; Souza and Oberauer, 2015), even if other explanations such as resource sharing sometimes were put in front as well (Oberauer et al., 2016). This inextricable situation is probably due to the fact that researchers adopted a dichotomous view of the problem because it is difficult to set apart the role of each explanation. After proving that one of the two explanations actually exists, it is often claimed that the other one is wrong. However, there are so many results in favor of the two explanations that it is likely that both exist. The challenge is now to design a working memory model that would describe the way time-based decay *and* interference operate and interact. This is the goal of the present paper.

Altmann and Gray (2002) proposed that decay and interference are related in such a way that when an item decays, it interferes less with future items. Decay is here viewed as the mechanism that creates distinctiveness between memory traces. They call it *functional decay*. Consider two items encoded at different times. The second one, when encoded, would appear distinct from the first one because of the decay of this item. Without decay, managing items appearing in close succession would be impossible because they would all be similarly active and hard to distinguish. This idea was also applied to model task switching (Altmann and Gray, 2002): decay reduces the cost of switching tasks because the new one always appears stronger than the previous one which has decayed since its encoding. However, as proposed by Oberauer et al. (2012b) in the SOB-CS model, another solution is to consider that a removal mechanism takes place to weaken the activation of previous items compared to the new ones. In any case, interference cannot be the sole phenomenon behind forgetting. It has to be supplemented by a process that tends to decrease the strength of previously encoded elements, whatever it is called functional decay or removal.

Altmann and Schunn (2002, 2012) incorporated these ideas in a computational framework based on the ACT-R architecture (Anderson and Lebiere, 1998). They first revisited Waugh and Norman (1965)’s data which were collected in a probe-digit experiment meant for weighting the respective effects of decay and interference. Participants were presented with a list of digits for study, followed by a probe digit which indicated that the memoranda following the first occurrence of the probe had to be recalled. This experiment was designed to measure the recall of digits as a function of the duration and number of memoranda following their presentation. Since there was a large effect of the number of interfering items and no effect of the presentation rate, the authors concluded that the main source of forgetting was interference. Altmann and Schunn (2012) first showed that the interaction, which was neglected by Waugh and Norman, appeared to be highly significant, which is in favor of a relationship between decay and interference. Then they designed a computational model that combines a decay equation and an interference equation. The first equation indicates that the base line activation (A) of an item depends on the time t since it was encoded:

A(t)=−0.5⁢ In(t)

The interference equation states that the probability *P*(*i*) of retrieving an item *i* depends on its activation *A*(*t*_i_) relative to the activation of other items *j*, weighted by a noise parameter s to model human variability:

$$P(i)=\frac{e^{\frac{A\left(t_{i}\right)}{s}}}{\Sigma_{j}e^{\frac{A\left(t_{j}\right)}{s}}}$$

When incorporated in the ACT-R architecture, the model was fitted to the Waugh and Norman (1965)’s data and showed that earlier items are recalled better in the fast presentation rate condition than in the slow one, indicating that time, and therefore decay, plays a role, in addition to a strong interference effect as well. In the same article, another simulation of the same model was carried out on an experiment using a Brown Peterson paradigm (Brown, 1958; Peterson and Peterson, 1959) and showed that decay itself is not enough to simulate the data.

Altmann and Schunn (2002, 2012) thus proposed a mathematical model that predicts a percentage of correct answers but does not describe the cognitive sub-processes involved, their time course and their schedule. For instance, it only describes reasons for forgetting but not the way the cognitive system is able to maintain memoranda throughout a trial by means of cognitive mechanisms such as refreshing (e.g., Johnson, 1992; Cowan, 2005; Barrouillet et al., 2011a), rehearsal (e.g., Vallar and Baddeley, 1982; Camos and Barrouillet, 2014) or consolidation (e.g., Bayliss et al., 2015; Ricker, 2015; De Schrijver and Barrouillet, 2017). In addition, although it is based on an existing cognitive architecture, several parameters had to be fitted to experimental data, which increases the risk of overfitting. For instance, five parameters had to be estimated to reproduce the 18 points of Waugh and Norman (1965)’s.

The present paper proposes to overcome these drawbacks by relying on a well-documented functional model of working memory, TBRS (Time-Based Resource Sharing model; Barrouillet et al., 2007, 2011a; Barrouillet and Camos, 2015). This model has a computational version, TBRS^∗^ (Oberauer and Lewandowsky, 2011) which simulates the so-called complex span task, in which memoranda interleaved with distractors are presented for serial recall. TBRS^∗^ implements processes such as encoding, retrieval/recall, refreshing and also simulates temporal decay. In line with the first strong version of the verbal TBRS model (Barrouillet et al., 2004), TBRS^∗^ only considers decay as the cause of forgetting. Following the more recent version of the TBRS model (Barrouillet and Camos, 2015), our aim is to extend TBRS^∗^ by supplementing it with an interference mechanism.

## Integrating Interference to a Time-Based Working Memory Model

In TBRS^∗^, which will be presented in details below, the only information stored in working memory is the set of associations between memoranda and positions. Throughout a simulated trial, connections between units from a memoranda layer and units from a position layer are created, weakened or reinforced according to where the attention is focused: new associations are created when a new memorandum is presented, they are reinforced when free time allows refreshing, but they decay when attention is devoted to another item or a distracting task. In TBRS^∗^, distracting tasks are not simulated *per se*, they are just considered as periods of time during which item-position associations decay. Therefore, TBRS^∗^ cannot simulate the way distractors interfere with memoranda.

### Time-Based Resource-Sharing (TBRS)^∗^

**FIGURE 1:**
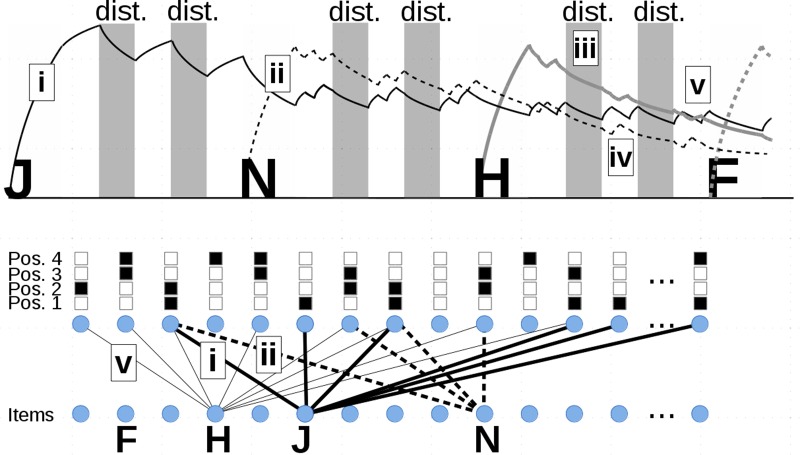
From Lemaire et al. (2017). **(Upper)** Simulated time course of a complex span task in which items J, N, H, and F are encoded (light gray areas). Two distractors are processed in-between each letter presentation (dark gray areas). Free time (white areas) is used to refresh items. Curves represent the total activation value of each item with respect to its position. **(Lower)** Connections between position units and item units. Each item is represented by a single unit. Each position is represented by several position units. Black and white squares represent position coding. For instance, position 1 is coded by units 3, 6, 8, 12, 13… Positions 1 and 2 share units 3 and 8. Examples of specific processes: (i) item J is encoded and associated to all units of position 1; (ii) item N is encoded and associated to all units of position 2; (iii) all activation values decay during distracting task; (iv) during free time, items are retrieved and refreshed for each position in turn; (v) during the free time following the second distractor after item H was presented, item H was erroneously retrieved at position 2 instead of N. Then, H was associated to all units of position 2.

We first present the basic TBRS^∗^ architecture on top of which we developed an interference mechanism. As we mentioned earlier, the TBRS^∗^ architecture is a two-layer network that connects item nodes to position nodes. Each item is represented as a single unit in the item layer, whereas each position is represented by a set of nodes in the position layer, such that adjacent positions share a particular proportion of nodes to simulate the usual confusion between adjacent positions (**Figure 1** from Lemaire et al., 2017). When a new memoranda is presented, connections are made between the corresponding unit in the item layer and position units in the position layer. When a distractor task occurs, all connections decay following an exponential law. When there is free time, all positions are considered in turn and for each one, a memorandum is retrieved and refreshed during a short period of time. This cycling process runs until another event appears such as a distracting task or a new memorandum presentation. Retrieving an item, whatever it is for refreshing or final recall, is done by computing the total activation for each item given the current position units. The retrieved item is the one with the maximal activation value, after a random Gaussian noise was first added to each value to simulate retrieval errors. **Figure 1** displays an example in which the task is to memorize items J, N, H, F for further serial recall, each one being followed by two distracting episodes presented every 2 s and each one processed for an average of 1 s.

Time-Based Resource-Sharing^∗^ is based on different parameters such as the encoding strength, the decay rate, the average duration of encoding, the standard deviation of the retrieval noise, etc. The duration of the processing task is usually estimated by the mean time taken by participants to respond to distractors. To model human variability, some parameter values are merely means from which the model draws a stochastic value. The details of these parameters are described by Oberauer and Lewandowsky (2011) and the main ones are presented in **Table 1**.

**Table 1:** Main TBRS^∗^ parameters.

Symbol	Default value	Meaning
R	6	Mean processing rate of encoding, refreshing, and recall
s	1	Standard deviation of processing rate
θ	0.05	Retrieval threshold
σ	0.02	Standard deviation of Gaussian noise added to item activations at retrieval
D	0.5	Decay rate
Tr	0.08	Mean time taken to refresh an item
Ta	Depends on the task	Mean duration of attentional capture by distractor processing steps


To sum up, as mentioned above, nothing is anticipated in TBRS^∗^ to simulate interference produced by distractors on memoranda. A way to solve that issue is to consider a distributed representation of items. As presented below, in our proposed model TBRS^∗^-I (for TBRS^∗^-Interference), memoranda and distractors are represented by set of units instead of single units.

### Time-Based Resource-Sharing (TBRS)^∗^-I

#### Working Memory Storage

Information stored in the TBRS^∗^-I working memory network is twofold. First, it is composed of associations between memoranda and positions as in TBRS^∗^. Second, it also includes position-independent representations of memoranda that are subject to interference. Actually, it is likely that these two kinds of information are stored: I could remember that a duck was presented in second position, but I could also remember that there was a duck somewhere without being able to indicate its position, or, conversely, that there was an animal in position 2, but being unable to recall which one.

Exactly like memoranda-position associations evolve during a trial, a memoranda representation may vary over time and especially at the critical moments of the task: at encoding, after a distracting episode or as the result of a refreshing process. At *encoding*, it is a representation coming from sensory inputs. It is considered as an intact representation of long-term memory knowledge (for instance, a horse). This idea can be hold in a unitary conception of memory proposing that working memory is the part of long-term memory activated above threshold (Engle et al., 1999; Lovett et al., 1999; Cowan, 2005) and also in a dual view of memory according to which the transient working memory representations result from the concatenation of perceptual information provided by the environment and elements stored in long-term memory (Logan, 1988; Barrouillet and Camos, 2015).

When a *distractor* occurs, the WM representation of the memoranda could be altered by this distractor (for instance, a lion which shares some features with a horse) by merging their units (e.g., Li, 1999; Conlin et al., 2005; Lewandowsky et al., 2008; Oberauer and Lange, 2008). The implementation of this interference phenomenon is presented below. Finally, when there is *free time*, a refreshing step may apply on this particular altered representation and tend to restore it to its initial long-term memory representation. Refreshing thus occurs after the degradation of memory traces by distractors for a restoration purpose (e.g., Barrouillet and Camos, 2015).

#### Distributed Representations

**FIGURE 2:**
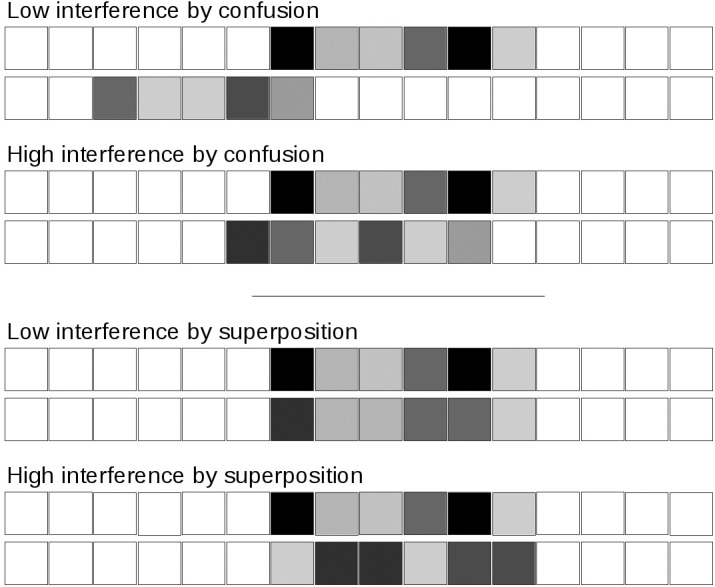
Illustration of various degrees of interference by confusion (resulted from unit overlap) and superposition (resulted from unit value similarity).

This section is devoted to the description of the modifications made on TBRS^∗^ to account for interference. It has been observed that similar items may interfere and produce distortions, for example when they share phonological features, but the opposite is also true: dissimilar items may interfere and lead to a detrimental effect because they mutually alter their representations (e.g., Oberauer et al., 2012a). By controlling both the overlap of units and the similarity of these units, it is possible to simulate these two different types of interference. Therefore, simulating various forms of interference can be achieved thanks to a distributed representation (Oberauer et al., 2016). We thus propose to model interference between memoranda and distractors by representing both of them in a distributed manner. Each one is then associated to a vector of units representing features encoded as numerical values, in a classical way (e.g., Mcclelland and Rumelhart, 1986). The number of units depends on the material of the experiment to be simulated. Sixty-four item units were used in all simulations of the present study but this arbitrary number could be easily extended if a higher number of features is necessary. Some features can be unused by a given stimulus but used by another one. For example, a black and white picture of a *dog* has no value on the phonological feature *T*, and no value on the visual feature *RED*. As we mentioned earlier, this representation can simulate the two opposite forms of interference that are found in the literature: interference could occur because of similarity or because of dissimilarity of memoranda and distractors (e.g., Oberauer et al., 2012a). We believe that this is because similarity is a vague concept that corresponds to different situations (see **Figure 2** in which values are represented in grayscale):

• If similarity means high feature overlap, then more similarity leads to more distortion because of interference by confusion (Oberauer et al., 2016);
• If similarity means low distance between the unit values that are common across the memoranda and the distractor, then more *dis*similarity will lead to more distortion because of interference by superposition (Oberauer et al., 2016).

For instance, there is more confusion between a letter and a word than between a letter and a spatial position because the first two overlap more than the last two. Therefore, the letter and the word interfere more. Similarity here leads to more interference. On the contrary, two items pertaining to the same domain (e.g., two non-words), *rame* and *zegg* (low similarity) would interfere more than *rame* and *vame* (high similarity) because there is more distortion when *rame* and *zegg* are superposed (Hoareau et al., 2017). Similarity here means less interference. Interference by confusion and interference by superposition can operate jointly (Oberauer et al., 2016). Both are represented in our model as shown in **Figure 2**.

In order to explain how TBRS^∗^-I works, the different processes are now presented in an almost chronological manner, along with an example.

#### Encoding

Encoding a memorandum in WM consists in retrieving its long-term memory representation (Barrouillet et al., 2011a) and binding this content (e.g., a letter) with a context (e.g., its current position, Oberauer and Lewandowsky, 2011; Oberauer, 2013). Consider a specific experiment in which five letters are displayed for serial recall and two distractors are presented in between each letter. Suppose letters J, N, and H and the two distractors after each letter have already been displayed. F is now presented. Its long-term memory representation is encoded into working memory exactly like in TBRS^∗^: connections between the units coding for item F and the units coding for position 4 are increased during 581 ms (drawn at random from the 500 ms default mean value). Right after encoding, there is free time before the next distractor appears. A refreshing process therefore applies, which will be presented later. Then a distractor appears.

#### Distractor Processing

Contrary to TBRS^∗^, distractors are items that also have a distributed representation. The model does not simulate the distracting task *per se*, but it describes the way the distractor affects the last-presented memorandum. Distractors are therefore encoded with a weaker strength than memoranda, to account for the fact that participants know whether an item is a memoranda or a distractor. That strength could have been estimated but we just set it to half the strength of memoranda.

In our example, F is altered by the distractor such that all its units that are shared with the distractor are slightly changed toward the distractor units. As a result, the working memory representation now differs from its long-term memory representation. For instance, F was represented by

**Table:** 

-	-	-	0.64	0.33	0.17	0.49	0.02	0.10	-	-	-	-


and the distractor was represented by:

**Table:** 

-	-	-	-	-	0.41	0.45	0.22	0.10	0.90	0.09	-	-


Item F and distractor are not represented along the first three units. The next two units are not affected because the distractor is not concerned by those features. The next four units of the memoranda are altered by a simple rule stating that new values are set halfway through the difference between the memoranda value and the distractor value (which corresponds to the average of the two values):

**Table:** 

-	-	-	0.64	0.33	0.29	0.47	0.12	0.10	-	-	-	-


The mean time during which a distractor impacts items stored in working memory is a parameter which is usually set to a value recorded in human experiments (i.e., the mean RT to perform the distracting task). As in TBRS^∗^, the effective duration is a random value drawn from that mean. During the process of a distractor, all items suffers from a temporal decay which is, as in TBRS^∗^, described by an exponential function. To sum up, in TBRS^∗^-I, two phenomena occur during distractor processing: a time-based decay of all memory items (as in TBRS^∗^) plus an interference-based degradation of the last-presented item as described in the current section.

#### Refreshing

During free time, memoranda are considered one at a time for refreshing purpose. The refreshing schedule is cumulative, exactly like in TBRS^∗^. Alternative schedules have been proposed (Portrat and Lemaire, 2015; Lemaire et al., 2017) but our goal here is to make minimal changes to TBRS^∗^ in order to be able to objectively evaluate the benefit of these changes. Numerous changes would make it difficult to understand the role of each one in the new behavior of the model. Future studies would test and specify refreshing schedules within such a new time- and interference-based model of working memory. For now, a cumulative schedule is used, which means that all positions are considered in turn, in a cyclical way. For each position, an item is considered for refreshing. This item corresponds to the working memory item which is the best associated to the current position. It is worth noting that it is not necessarily the one which was encoded at that position because of retrieval errors that will be presented later in the *retrieval* section.

Once an item is retrieved, refreshing operates in two ways: it reactivates the association between the item and its context and restores its representation that has been damaged by distractors. *Reactivation* means increasing its associations to the current position that faded away because of time-based decay. This idea is based upon a reactivation conception proposed elsewhere: attentional maintenance in working memory counteracts forgetting by reactivating memory traces (Cowan, 1988; Barrouillet et al., 2004; Oberauer and Lewandowsky, 2011; Portrat et al., 2015). Moreover, the fact that the reactivation process applies on the associations between items and their context is also supported by previous studies according to which refreshing strengthen the binding of items to their serial positions in a list (Oberauer, 2002; Loaiza and McCabe, 2012). In such a view, when refreshing is possible, the focus of attention is directed on a given item for binding strengthening (Oberauer and Hein, 2012; Oberauer, 2013). Additionally, r*estoration* means repairing the working memory representation that has been affected by distractors. This restoration sub-component of refreshing is in line with the conception by which forgetting results from a degradation of the transient working memory representations and refreshing aims at reconstructing the mental representations to become as close as possible to their original form (Barrouillet and Camos, 2015). The ways these two refreshing sub-processes are implemented in TBRS^∗^-I are now detailed.

First, refreshing reinforces the connections between the retrieved item (here, J) and the current position units (here, units coding for position 1) following the same function as the one used in initial encoding, but with a much shorter duration. This duration is 80 ms as in the original TBRS^∗^ implementation (parameter *Tr*) although recent experimental or computational findings proposed a shorter duration of about 40–50 ms. For instance, Camos and Barrouillet (2014) designed an experiment in which participants could postpone the processing of a distractor and observed that this postponement was about 40–50 ms times the number of memoranda to maintain. Jarrold et al. (2011) found a linear trend between the distractor response time and the position of this processing episode in the memory list and estimated the refreshing duration to about 40 ms. Vergauwe and Cowan (2015) also found that refreshing would take about 40 ms.

The second purpose of the refreshing process is to restore the representation of the memoranda by modifying its working memory representation using the closest long-term representation. Back to our example, let us make a distinction between J_LTM_, which is the engram of J in long-term memory, and J_WM_, which is the representation of J in working memory at a given time, possibly distorted by distractors. After J_WM_ was retrieved for position 1, the closest stable long-term representation is identified. If J_WM_ was not distorted too much by distractors, it is likely that J_LTM_ is the closest one. Refreshing J_WM_ is then moving each of its units toward the J_LTM_ units, by the same mechanism presented before: each J_WM_ unit value is set to the average between itself and the corresponding J_LTM_ unit value. The refreshing of working memory representation and the interference induced by distractors are therefore based on the same basic process, except that the first one is beneficial and the second one is detrimental. When a memoranda is refreshed, its units are modified toward the stable LTM units (for each memoranda unit *U*, *U*_WM_ = ½(*U*_WM_ + *U*_LTM_). In the same way, when a memoranda is altered by a distractor, its units are modified toward the distractor units (for each memoranda unit *U*, *U*_WM_ = ½(*U*_WM_ + *U*_dist_).

#### Retrieval

Retrieving an item consists in selecting the one which is the best associated to a given position. Each item, memoranda or distractor, even those that have not been presented in the current trial, are candidates. The process is twofold. First, the sum of the connections between each item and the units coding for the current position are computed in order to select the highest value. Before that decision, a random noise value is added to each sum in order to simulate retrieval errors. That part is similar to TBRS^∗^. However, the retrieved item is a working memory object that could have been altered from its initial representation coming from long-term memory, because of interference. Therefore, the second step is to identify to which long-term memory representation that object corresponds, in order to output a stable and not a distorted object. The long-term representation that is selected is the one which is the closest to the working memory object that has been retrieved, by computing a numerical distance (actually, a simple mean square error) between distributed representations.

#### Recall

Recall is viewed as a sequence of retrieval processes, one for each position in turn. It is worth noting that decay applies during recall, which means that the last items suffer more from time-based decay than the first items because they are retrieved later. Interference may also occur during the recall step, because an item just recalled may interfere with the still to-be-recalled ones, but this is outside the scope of the present study which is only concerned by item-distractor interference. Further studies will integrate this aspect.

**FIGURE 3:**
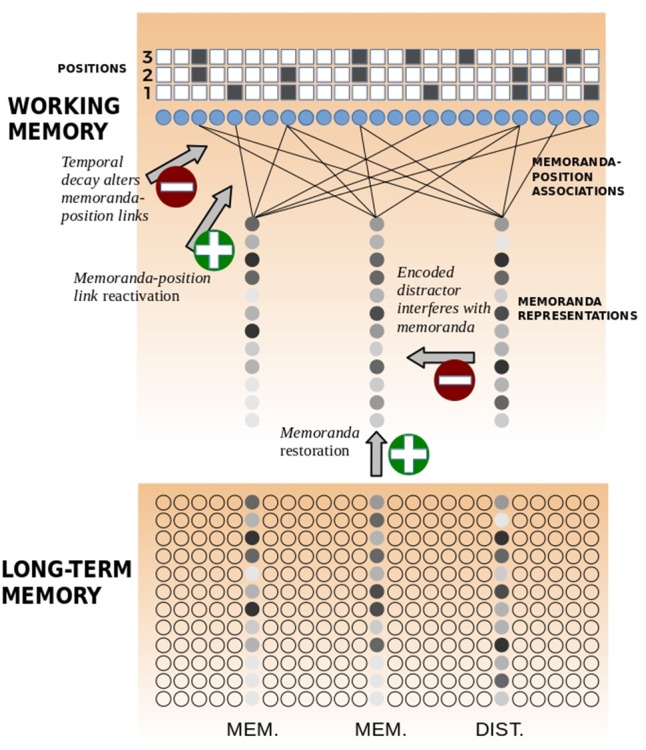
TBRS^∗^-I architecture.

To sum up, **Figure 3** summarizes the main features of the TBRS^∗^-I architecture. It is composed of a working memory and a long-term memory. Memoranda and distractors are encoded in working memory by means of association links to position units. For instance, **Figure 3** shows that the first memoranda (the one on the left) is associated to position 1 (units 5, 8, etc.). Suppose that item 1 was not altered by a distractor: it has the same representation as the one in long-term memory. The second memoranda (the one in the middle) is associated to position 2, which shares some units with position 1. The third item (the one on the right) is a distractor which is associated to position 2 as well. Therefore, this distractor interferes with item 2 and modifies the representation originating from long-term memory. Another detrimental phenomenon, namely temporal decay, decreases memoranda-position associations. Maintenance mechanisms are also illustrated: when there is free time, refreshing increases the memoranda-position associations (called *reactivation*) but also repairs the memoranda representation by means of the long-term memory representation (called *restoration*).

## Testing the New Model

In order to test the relevance of the TBRS^∗^-I model, we relied on the benchmarks proposed by Oberauer et al. (2016). These benchmarks are strong findings that have been observed and replicated in the literature. They were grouped into three classes: set-size effects and their modulation by heterogeneity of the memoranda set, distractor effects modulated by the memoranda-distractor interference degree and individual differences. Our study is only concerned with the second group (distractor effects) because, for the moment, our current model neither implements interference within the memoranda set, nor individual differences. There are six findings in that group:

• B1. Recall performance decreases as the cognitive load (CL) of the task increases, where cognitive load is defined as the proportion of time during which attention is captured by the distracting activity (Barrouillet et al., 2004). For instance, if the distracting task is composed of several sub-tasks capturing attention for a second, each one followed by 2 s of free time during which people can refresh memory traces, the cognitive load would be 1/3 (1 s of attentional capture every 3 s).
• B2. Duration of distractor processing affects performance only if distractors differ from each other. Duration of repeated distractors has no effect on performance.
• B3. Duration of a retention interval without any distracting task impairs performance.
• B4. Performance is more affected by distractor belonging to the same content domain as the memoranda than by distractors from another domain.
• B5. Performance is, however, also affected – albeit less strongly – by distractors from a different domain.
• B6. Within the same domain, performance is more affected when distractors belong to the same category as the memoranda than when they belong to different categories.

**FIGURE 4:**
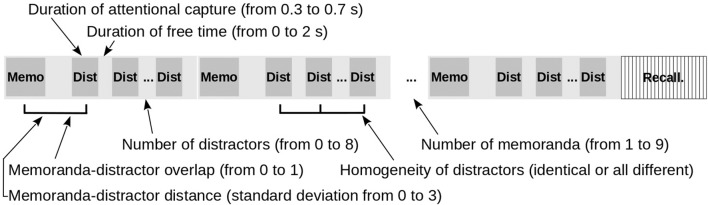
Variables manipulated in the simulations of a complex span task.

In order to test whether our new model extension is able to replicate these benchmark findings while not sacrificing its previous behavior, we performed the same set of simulations proposed by Oberauer and Lewandowsky (2011, simulation 1) to test TBRS^∗^, but we added three variables to manipulate the degree of interference between memoranda and distractors: two additional variables to manipulate the interference by confusion and by superposition and an additional variable to manipulate the homogeneity of distractors. The six following variables were then defined (**Figure 4**):

• ND: Number of Distractors in each burst following each memorandum (0, 1, 4, and 8);
• DAC: Duration of Attentional Capture by each distractor (0.3 s, 0.5 s, 0.7 s);
• FT: Free time following each distractor processing, during which attention can be devoted to refreshing (0, 0.1, 0.6, 1.2, and 2.0 s);
• MDO: Memoranda-Distractor Overlap operationalized by the percentage of unit overlap between memoranda and distractor representations (0, 0.2, 0.4, 0.6, 0.8, 1) to manipulate interference by confusion (see **Figure 2**, upper panels);
• MDD: Memoranda-Distractor Distance computed as the standard deviation of the distance between memoranda and distractor units (0, 0.5, 1, 1.5, 2, 2.5, 3) to manipulate interference by superposition (see **Figure 2**, lower panels);
• HOD: Homogeneity of distractors (all identical or all different).

The recall score of a trial is the percentage of memoranda correctly recalled. Following Oberauer and Lewandowsky (2011), the number of memoranda was also varied from 1 to 9 memoranda and the results summed to obtain a span score. All combinations were tested, which represents 4 × 3 × 5 × 6 × 7 × 2 × 9 = 45,360 different cases, with 5,000 runs for each case.

All other parameters, such as processing rate, noise, decay rate, threshold for retrieval, were set at the default values proposed by Oberauer and Lewandowsky, (2011, see **Table 1**). It is worth noting that our approach does not require to estimate the values of some free parameters as it is often the case in cognitive computational modeling: the model was implemented according to the theoretical proposal, ran with the appropriate values for variables and the recall performance directly analyzed. We now present the results of all simulations, with respect to the benchmark findings listed previously.

### Cognitive Load Effect (B1)

**FIGURE 5:**
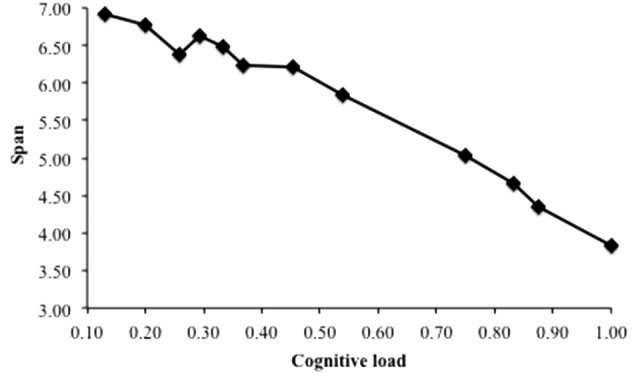
Span predicted by TBRS^∗^-I as a function of the cognitive load.

**Figure 5** presents the span performance as a function of the cognitive load of the task, computed as DAC/(DAC + FT). Data were averaged across all other variables. The three values for the duration of attentional capture and the five values for the free time gave 12 distinct cognitive load values. The lowest value corresponds to the case when attention is captured by the distractors during 13% of the time of the distracting phase. The highest value of 1 is the case when there is no free time: attention is fully captured during the distracting phase. Results replicate a strong relationship between cognitive load and recall performance, as was observed several times in the literature (Barrouillet et al., 2004, 2007, 2011a). For instance, Barrouillet et al. (2011a) conducted a meta-analysis from several experiments using complex span tasks in which the processing component involved executive functions such as updating, inhibition, response selection and retrieval leading to different cognitive load values. They found a linear relationship between the mean memory span of participants and the cognitive load regardless of the nature of the processing component. This relationship was also predicted by the original TBRS^∗^ computational model (Oberauer and Lewandowsky, 2011) and its recent extensions (Portrat and Lemaire, 2015; Lemaire et al., 2017). This was expected because increasing the cognitive load means less time for refreshing items and more time for temporal decay. Therefore, memoranda are more likely to be forgotten and the recall performance decreases consequently. Supplementing TBRS^∗^ with a component able to model the interference mechanisms did not affect its ability to reproduce the robust cognitive load effect.

### Homogeneity of Distractors (B2)

**FIGURE 6:**
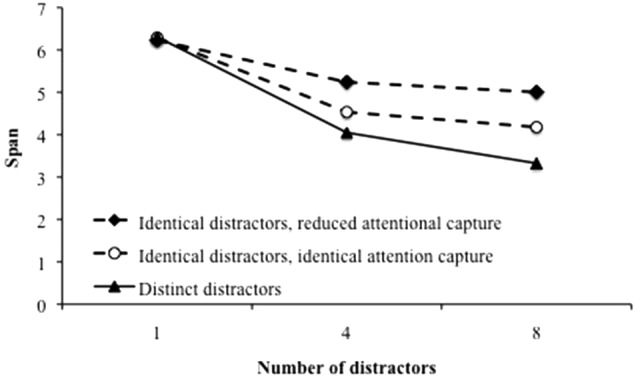
Span as a function of the number of distractors for distinct distractors, repeated distractors all processed with the same duration and repeated distractors processed faster.

Benchmark B2 contrasts distinct vs. repeated distractors within a trial. It says that the duration of processing distractors has an effect on performance only if distractors are distinct from each other. This prediction is supported by several experiments. For instance, Lewandowsky et al. (2010) asked participants to remember letters in between which they had to read aloud series of words. As expected, recall performance decline with the number of words to utter, from 0 to 1 and 3, because the duration of the retention interval affects performance. However, when four repeated words were presented, recall performance was not different from the case of a single one, although the duration of the retention interval was longer. McFarlane and Humphreys (2012) replicated the result when comparing situations with a single pair of distractors to be pronounced (dog–cat), a repeated pair of distractors (dog–cat, dog–cat) and a double pair of distractors (dog–cat, rat–cow). Lewandowsky et al. (2008) as well as Barrouillet et al. (2013) obtained a similar finding, although distractors were presented during the recall phase. However, it seems that this finding is only observed if the cognitive load is kept constant. In fact, when the balance between the duration of distractor processing and free time is manipulated, longer duration of processing bursts of repeated distractors may have an effect on performance if cognitive load increases at the same time (Plancher and Barrouillet, 2013). In order to test benchmark B2, variable HOD was considered since half of the simulations were performed with distractors that were all different from each other throughout a trial and the other half with repeated distractors within a trial. The appropriate variable to reproduce a variation of the duration of processing distractors irrespective of the cognitive load is ND, the number of distractors. All other things being equal, varying the number of distractors induces by itself a variation of the duration of processing distractors. As usual, at first glance, data were averaged over all other variables. **Figure 6** displays the span as a function of the number of distractors, for the two conditions (i.e., repeated vs. different distractors; lower curves). As observed in humans, duration of processing distractors has a strong effect on performance when distractors are all distinct. However, simulations also show an effect, albeit smaller, when distractors are repeated which is not what benchmark B2 predicted. One of the reasons of this discrepancy could be that the simulations did not appropriately reproduce the human processes. In fact, Plancher and Barrouillet (2013) proposed that the duration of attentional capture is weaker when distractors are repeated because subsequent occurrences of a given distractor require less resource to manage. However, in our simulations, the duration of the attentional capture by distractors is the same whatever distractors are distinct or repeated. We then modified the computational model to spend less time processing repeated distractors than processing distinct distractors. Except for the first distractor of each burst, all repeated distractors were processed by the model with a very short duration (50 ms). **Figure 6** shows the results (upper curve). Performance becomes higher but the effect of distractor duration is still observed.

**FIGURE 7:**
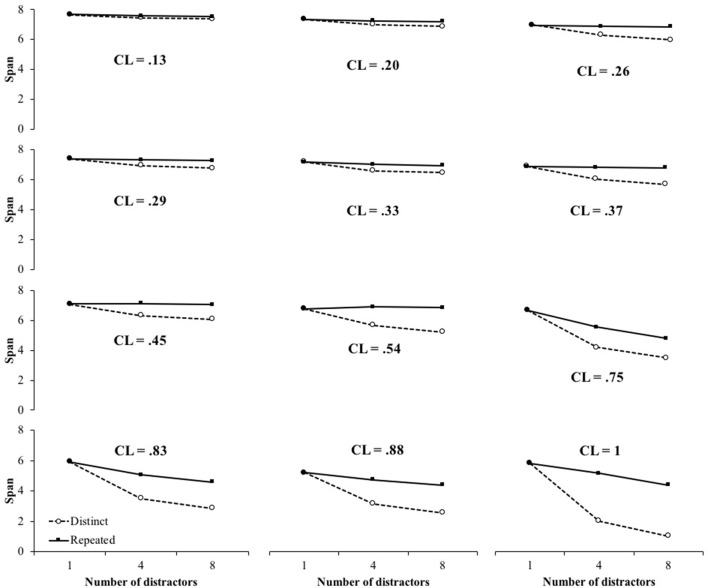
Span as a function of the number of distractors for distinct distractors and repeated distractors with a reduced attentional capture for different cognitive loads. In the repeated condition, actual CL are lower than what is indicated because of the reduced attentional capture.

However, as stated above, the effect of the duration of the distractors has been shown to depend on the temporal conditions of the processing task (Plancher and Barrouillet, 2013). **Figure 7** displays the effects of the number of distractors for distinct and repeated conditions, under different CL. For very low CL, there are no effects, whatever distractors are distinct or not. For the higher CL, there is a number of distractor effect in both conditions, in the range of number of distractors which was studied. However, for intermediate CL, there is an effect when distractors are distinct and no effect when distractors are repeated. TBRS^∗^-I therefore reproduced the benchmark B2 for certain cognitive load values. The reason why this benchmark is only reproduced for some values of cognitive load is now discussed.

Actually, if the cognitive load is sufficiently high, refreshing does not compensate decay and an effect of the number of distracting operations can be observed (Oberauer and Lewandowsky, 2011). However, adding more and more distractors has a detrimental effect on performance up to a point where refreshing and decay are in equilibrium and adding more distractors would not make any difference. At this point, some memoranda have been forgotten and this forgetting enables refreshing to act on a reduced remaining amount of information. As a consequence, the effect of the number of distractors that can be observed in conditions with relatively few distractors (say, 1 vs. 4) disappears in conditions with a higher number of distractors (say, 4 vs. 8). The relationship between number of distractors and memory performance is therefore not always linear. **Figure 7** shows precisely this point: the effect of the number of operations exists or not depending on the cognitive load and the number of distractors considered. Therefore, the difference of cognitive load when distractors are repeated or not causes a differential effect of the number of distractors across conditions.

This analysis shows that experimental studies supporting B2 were appropriately designed to define a distinct distractor condition able to show an effect of the number of operations, and a repeated distractor condition with no effect of the number of distractors. For instance, consider the two curves of **Figure 7** with a cognitive load of 0.54. Benchmark B2 is verified between four and eight distractors: there is an effect of the number of distractors when they are all distinct, but no effect when they are repeated. However, it is not the case for a cognitive load of 0.75 between four and eight distractors: there is an effect in the two conditions. There is thus a range of CL and number of distractors where the benefit of repeated distractors is enough to compensate the strong detrimental effect of high cognitive load situations. Actually, our model implements a more complex detrimental/beneficial balance than the simple decay/refresh one, because it involves two detrimental mechanisms (decay and interference) as well two refreshing mechanisms (reactivation and restoration). However, it is able to reproduce the B2 benchmark in specific, albeit not extreme, conditions.

**FIGURE 8:**
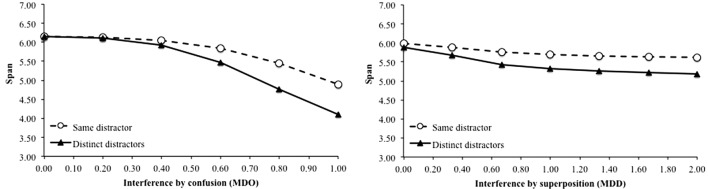
Span as a function of the level of interference by confusion, implemented by the percentage of overlap between memoranda and distractors **(left)** and as a function of interference by superposition, implemented by the standard deviation of the distance between memoranda and distractors **(right)**.

Moreover, it is worth noting that the studies behind this benchmark are based on the idea that the better performance when distractors are repeated is because they create less interference with memoranda. Our computational model is a powerful tool to investigate that hypothesis by measuring the effect of each kind of interference on the recall performance. Interference by confusion is studied by manipulating the degree of overlap between memoranda and distractors. A burst of a unique repeated distractor would always affect over and over the same part of memoranda, leading to less interference than different distractors and then higher performance. Interference by superposition is studied by manipulating the distance between memoranda and distractors. Repeated distractors would affect the memoranda units by making them resemble their units but the effect would tend to decrease with each additional distractor because it is always modified in the same manner. In order to observe whether our model would replicate these different interference effects, we varied the memoranda-distractor overlap and the memoranda-distractor similarity, respectively. The left panel of **Figure 8** shows that interference by confusion seems to play a role in the fact that repeated distractors lead to better recall performance, but mainly for high interference values. The right panel of **Figure 8** shows a similar pattern for interference by superposition with benefit of the “same distractors” condition compared to the “different distractors” condition. It also reproduces the detrimental effect of the dissimilarity between distractors and memoranda (Oberauer et al., 2012a; Hoareau et al., 2017). It is worth noting that this simulated effect is not massive. To sum up, the beneficial effect of a unique repeated distractor could be due to a lower attentional capture, as shown by the increased memory performance when attentional capture is artificially reduced (**Figure 6**, upper curve and **Figure 7**). However, this effect could also be due to less interference when there is only one distractor to process (Oberauer et al., 2012a).

### Duration of an Unfilled Retention Interval Impairs Memory (B3)

**FIGURE 9:**
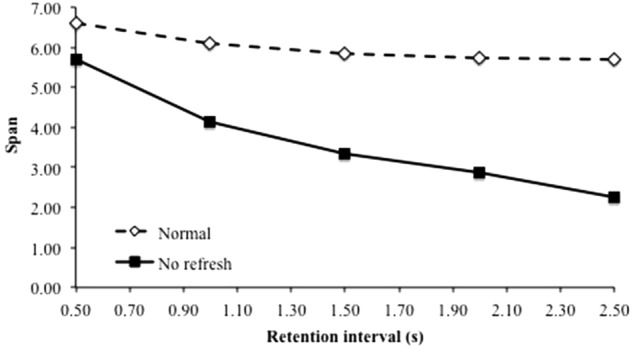
Span as a function of the duration of the retention interval without distractors, following the encoding of each memoranda.

Benchmark finding B3 concerns the case of visual or spatial memoranda for which some studies have found that extending the duration of the retention interval without any distractor processing impairs memory. The original TBRS^∗^ model as well as our model cannot reproduce that finding because during a retention interval without distractors, these models perform a refreshing process that could maintain the memoranda. The upper curve of **Figure 9** displays span scores as a function of the duration of the retention interval following each presentation of a memorandum. The slight loss of performance in this normal condition is probably due to high interference conditions where refreshing does not exactly compensate forgetting, leading to a higher probability of forgetting as time goes on.

However, the B3 findings were obtained with visual or spatial memoranda for which participants were unable to set up a refreshing process. For example, Ricker and Cowan (2010) found this effect using unconventional visual characters but observed no forgetting with English letters. They suggested that the reason lies in the fact that unconventional characters do not form a single, identifiable chunk in long-term memory and, as a consequence, cannot be refreshed. We then designed a specific version of our model in which the refreshing process was disabled. The lower curve of **Figure 9** shows the performance of that specific model as a function of the duration of the retention interval following the encoding of each item. Extending the retention interval impairs memory as predicted.

### Domain-Specific Effect of Processing (B4)

B4 groups the binary findings that processing distractors from the same content domain as the memoranda leads to a larger impairment than when the domains are different. This reflects an effect of interference by confusion that occurs when the representations of memoranda and distractors share the same features and therefore can be harder to distinguish from each other. When averaging all data with no overlap at all (MDO = 0), which corresponds to the case of distractors from another domain than memoranda, the mean span is 6.15. The span is 4.49 when averaging all cases where distractors and memoranda share the same units, which is a 100% overlap (MDO = 1). These results are in line with the B4 findings: the domain of distractors has an effect on memory performance with poorer memory when distractors belong to the same domain compared to distinct domains. Contrary to TBRS^∗^, TBRS^∗^-I is thus able to reproduce an effect specific to interference-based forgetting.

### Cross-Domain Impairment of Memory by Processing (B5)

**FIGURE 10:**
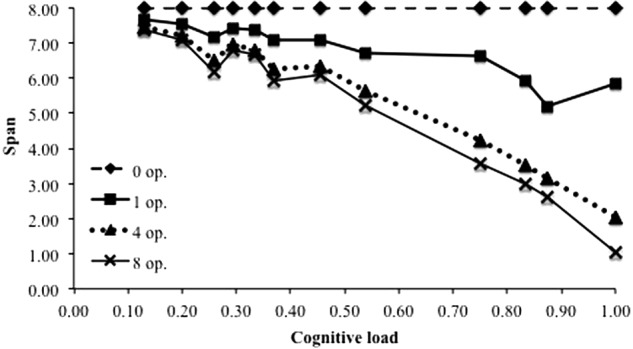
Span as a function of the cognitive load for tasks without distractors and tasks with 1, 4, or 8 distractors, when memoranda and distractors belong to different domains (i.e., when MDO is set to zero).

As just discussed, memory is impaired when memoranda and distractors belong to the same content domain. However, findings B5 say that memory is also impaired, albeit slightly, when memoranda and distractors belong to different domains. Only the data with no memoranda-distractor overlap (MDO = 0) were then considered. **Figure 10** shows that even if memoranda and distractors belong to different domains, distractors impair memory. This effect of the number of distractors appears dependent on the cognitive load, as we discussed earlier. Indeed, the effect is stronger for higher cognitive load values as already shown regarding the B2 benchmark. The classical pattern observed in TBRS^∗^ by Oberauer and Lewandowsky (2011) is then replicated in TBRS^∗^-I.

**FIGURE 11:**
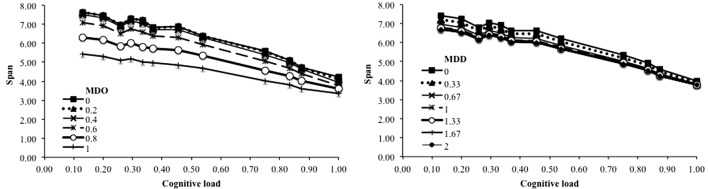
Span as a function of the cognitive load for different degrees of MDO **(left)** and for different degrees of MDD **(right)**.

As an attempt to reconcile decay and interference theories of forgetting, an interesting study is to investigate the interaction between cognitive load and degree of interference. Cognitive load is the key demonstration of proponents of the decay-based explanation of forgetting but most of the experiments showing a cognitive load effect were designed to minimize interference between memoranda and distractors (e.g., Barrouillet et al., 2011a, but see Vergauwe et al., 2009, 2010). The left panel of **Figure 11** shows the cognitive load effect for various degrees of interference by confusion. The cognitive load effect decreases as a function of the degree of interference. While being less marked for the higher values of interference, the cognitive load effect is always observed: even for a full overlap between memoranda and distractor, higher cognitive loads lead to worse memory performance. A similar result was obtained with the interference by superposition, showing that the cognitive load effect exists whatever the degree of interference between distractors and memoranda (**Figure 11**, right panel).

### Heterogeneity Benefit (B6)

**FIGURE 12:**
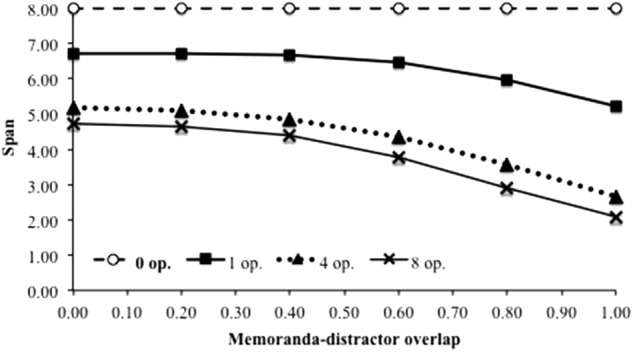
Span as a function of memoranda-distractor overlap for 0, 1, 4, or 8 distractors.

Benchmark B6 concerns the case of distractors and memoranda from the same domain, which is represented in our model by a non-zero memoranda-distractor overlap (MDO > 0). The finding is that distractors pertaining to different classes than the memoranda (e.g., words and digits) impair memory less than distractors from the same class (e.g., words and words). This can be studied by manipulating the degree of overlap MDO: distractors and memoranda from the same class share more units than distractors and memoranda pertaining to different classes because, in this latter case, some units of memoranda are not affected at all by distractor units. **Figure 12** shows that performance depends on the degree of memoranda-distractor overlap: whatever the number of distractors, more interference by confusion leads to worse performance, in a non-linear way. TBRS^∗^-I is then able to reproduce a human behavior that is specific to interference-based forgetting, which was not possible with TBRS^∗^.

## Discussion

Time-Based Resource-Sharing^∗^-I is a computational model of working memory which includes both decay-based and interference-based mechanisms of forgetting. It is supported by the solid TBRS verbal model in which attention plays a central role by considering that an individual memory trace decays all the time except when attention is focused on it, either during its encoding or during short refreshing episodes that could occur during free time (Barrouillet et al., 2004). TBRS has been tested numerous times in the literature and constitutes a strong cognitive description on top of which a computational model can be designed. TBRS^∗^-I also relies on the basic architecture of TBRS^∗^ (Oberauer and Lewandowsky, 2011), a TBRS computational implementation which has also been tested several times after its first publication (Lewandowsky and Oberauer, 2015; Portrat and Lemaire, 2015; Hoareau et al., 2016; Lemaire et al., 2017). In this section, we will discuss whether TBRS^∗^-I is compatible with a unitary or dual view of memory but also the issue of integrating time and interference in a single model as well as the parsimonious and reversible mechanisms that we propose.

### Unitary or Dual View of Memory?

In the present implementation of a WM model covering both decay-based and interference-based forgetting, we decided that the human cognitive system would include at least two instances of a given information, one for ongoing thoughts managed by the working memory system and at least another one stored in long-term memory. This choice has been driven by a non-unitary view of memory (see Kintsch et al., 1999 for a review) that was recently favored by the authors of the original TBRS model (Barrouillet and Camos, 2015). In such a framework, working memory serves for the integration of information from the perceptual environment with elements from long-term memory and the refreshing mechanism aims at preserving and reconstructing the degraded working memory traces. However, a more continuous relationship between working memory and long-term memory is postulated by other researchers proposing that working memory is the activated portion of long-term memory (see Kintsch et al., 1999 for a review). From a computational point of view, this unitary vision could be described by supplementing each long-term memory item unit with a degree of activation which would change as a function of the state of the item. Therefore, there would be only one instance of each item instead of two. Each unit would be twofold and composed of a stable long-term value to implement similarity between items and an activation value to implement its memory state. For instance, the unit *dangerous* of the item *shark* would have the long-term value 0.8 (because sharks are dangerous animals; *rabbit* would have instead either no or a very low value) and may have the activation value 0.9 if the item *shark* was recently encoded but only 0.2 if this item is about to be forgotten or has not been presented in any memory list. In this view, there would be no specific set of items representing working memory. Instead, working memory would be viewed as all units with sufficiently high activation values.

Accordingly, considering interference within a unitary or a dual view of memory is probably just a matter of implementation. Similarity-based interference is implemented in the present dual-view model such that interference is a modification of the memoranda original value to bring it closer to the distractor value. In the same way, refreshing is modifying the memoranda degraded value to bring it closer to the original long-term memory value. It is also possible to implement these two mechanisms within a unitary view of memory: interference would be, in that case, a decrease of the memoranda activated value as a function of its similarity with the interfering distractor and refreshing would be an increase of the deactivated value toward the highest value. In fact, the amplitude of these variations of activation values would be a function of the similarity between memoranda and distractors. It is therefore about the same to consider that a distractor unit affects a memoranda unit to make it at his image or to modify its activation value according to their similarity. Hence, considering either memory as a unitary or a dual system does not preclude an integration of the two sources of forgetting in a single model.

### Interference and Time in a Single Model

Another strong and longstanding debate in the literature concerns the source of forgetting opposing time-based and interference-based explanations. This debate was in need of an integration proposal. Following several attempts (Altmann and Schunn, 2002, 2012), we showed in this paper that a working memory decay-based model can be supplemented with a mechanism to deal with interference. The key of that integration was to consider a dual role for decay and refreshing, each one operating on the memoranda levels of activation as well as on the memoranda representations. Decay is both time-based and interference-based. Time-based decay operates on the activation levels of associations between memoranda and their contexts by decreasing them as time goes on. Interference-based decay operates on the memoranda representations by having them altered by distractors. In the same way, we showed that refreshing, which is one of the maintenance mechanism of decay-based models, can be also used to model the cognitive processes that counteract the detrimental effect of interference.

As originally proposed in TBRS^∗^, refreshing counteracts time-based decay through the reactivation of the position-memoranda links (e.g., Oberauer and Lewandowsky, 2011). The original TBRS^∗^-I proposal is to add a restoration component to refreshing which counteracts interference by reinstating the memoranda representations that have been damaged by distractors, relying on the stable long-term representations to do so. Although this conception of refreshing is novel from a computational perspective, it echoes a theoretical proposal that was missed in the original TBRS^∗^ model (e.g., Barrouillet and Camos, 2015). We thus propose that refreshing is twofold: it operates on the position-memoranda levels of activation, by increasing them, but also on the memoranda representations, by restoring them. As an illustration, suppose your nephews have build a nice sand castle on the beach and you want their parents to see it when they come back at the end of the day. First, you have to memorize the location of the castle on the large beach. Second, you have to maintain the castle in a good shape because other “distracting” children may transform it to their taste. You should therefore operate on the link between the castle and its location but also on the representation of the castle itself. Both are independent from each other but both are necessary. Otherwise, you could end that day with a pretty good sand castle that you could not retrieve, or you may remember where was the castle but it was unfortunately altered by “distracting” children.

**FIGURE 13:**
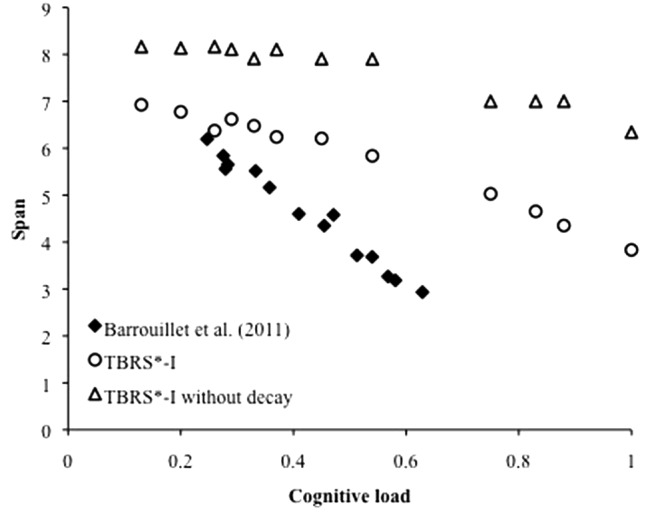
Span as a function of the cognitive load, for the Barrouillet et al. (2011a) meta-analysis, the TBRS^∗^-I model and the same model without any time-based decay.

Our proposal is based on the assumption that decay and interference both exists, because of numerous findings in the literature on both sides. However, it is tempting to use the model to check whether a sole explanation would be sufficient, by disabling one or another component. Disabling the interference mechanisms has already been performed because it corresponds to the TBRS^∗^ model. Disabling the decay mechanisms is, however, innovative in such a framework. We therefore ran the exact same simulations presented before with a decay rate set to zero. We also ran these simulations on another version of the model in which the reactivation process counteracting decay was also turned off. Actually, both simulations provide the same results, probably because reactivation is useless when memoranda/position associations do not decay. We chose the cognitive load effect as a ground truth because it is a massive effect that is recognized by both decay and interference proponents. Critically, simulations showed no cognitive load effect on recall performance for values of the cognitive load lower than 0.6. Of particular importance is that these values correspond to the range of cognitive load values reported in the literature testing the cognitive load effect. In fact, we compared the outcomes of the simulations to a reference paper (Barrouillet et al., 2011a), already mentioned in Section “Cognitive Load Effect (B1),” that gathered various experiments with different cognitive load values. This paper showed a linear relationship between cognitive load and performance estimated as *Span = -8.33 CL + 8.13*. **Figure 13** shows this linear relationship as well as the data of our two models, TBRS^∗^-I and TBRS^∗^-I without decay. The two models have a general performance higher than the human data, probably because of a discrepancy between materials used for human testing and simulations. Actually, we could not precisely reproduce the material of the human experiment and its uncontrolled interference effects. Reducing the overall model performance could be done with a higher memoranda-distractor overlap for example. However, we are interested in reproducing the effects rather than the magnitude of performance. Although our initial model TBRS^∗^-I does not show a cognitive load effect as strong as the one reported in humans by Barrouillet et al. (2011a), there is still a decrease of memory performance with increased CL which does not exist when decay is set to 0. It is therefore likely that temporal decay plays a role in working memory.

However, for higher values of the cognitive load, the model without decay shows a drop of performance, although there is still no effect when the cognitive load increases from 0.75 to 0.88. It would be difficult to draw any strong conclusions about this result because we could not find any human data for these high values of cognitive load. However, it is interesting to note that a pure model of interference is still able to account for the cognitive load effect under specific conditions. The reason is that when there is more free time, the restoration process has more time to counteract the detrimental effect of distractor interference. However, there is an underlying hypothesis that restoration depends on time. If our mechanism were not dependent on the duration of the free time, there would be no cognitive load effect at all. The only way to show a cognitive load effect if decay does not exist is that the reverse mechanism does depend on time. The removal mechanism that was proposed by Oberauer et al. (2012b) as part of the SOB-CS model relies on a similar assumption: removal takes time (Oberauer, 2001). Hence, the cognitive load effect can be somehow observed under the no decay hypothesis, to the extent that time plays a role in a restoration process. However, because that was not our goal, our results do not resolve the question of the existence of decay. Rather, our contribution is an integration of mechanisms that have been often considered separately, considering that there are strong evidence for both an interference and a decay explanation.

### A Single Mechanism to Interfere and Restore

The interesting proposal of our model is that it is exactly by the same mechanisms that distractors alter memoranda (by interfering or dissociating from the context) and long-term representations reinstate working memory memoranda (by restoring the representation or reactivating its links with the context). In a sense, the additional *restoration* sub-component of refreshing a working memory representation can be viewed as the result of an interference with its long-term representation.

During the design of TBRS^∗^-I, we paid attention to add a limited number of parameters, because the more parameters, the easier the fit to a given behavior. Actually, only one free parameter was added: distractors are encoded with half the strength of memoranda. This value was not estimated and thus set arbitrarily but it might be interesting in the future to assess more precisely that value. The degree of overlap between memoranda and distractors as well as the similarity between them are not free parameters: they have to be set according to the material used in the experiment that has to be reproduced. The only modifications compared to TBRS^∗^ concern the distributed representation of items and memoranda and the mechanisms that implement interference-based decay and the *restoration* component of refreshing in addition to the existing time-based decay and the *reactivation* component of refreshing that were not modified. From a computational point of view, it is worth noting that alteration by interference and repairing of memoranda after interference are implemented by the same simple averaging mechanism.

Another model, SOB-CS (Oberauer et al., 2012b), has also a reversible mechanism: to counteract forgetting due to distractors, a removal process operates right after a distractor has been processed. Removal directs attention toward the trace of the distractor in order to progressively suppress it from the common working memory representation shared by memoranda and distractors. Removing a distractor is performed in the exact inverse manner it was encoded. However, removal has been criticized (Barrouillet and Camos, 2015; Hoareau et al., 2017). Notably, this mechanism assumes that people would maintain information in mind by directing their attention elsewhere, i.e., precisely at the information that are supposed to distract them from memoranda. We rather propose that the free time available to restore a memoranda does not consist in removing the distractor, but rather repairing the memoranda through the restoration component of refreshing. We consider thus that, during that free time, attention is focused on the memoranda to be repaired rather than on the distractor to be removed.

Our computational model then showed that the TBRS verbal model easily handles an interference mechanism, which is what its authors claimed (Barrouillet and Camos, 2015). However, most experimental studies within TBRS manage to minimize similarity-based interference between memoranda and distractors by often opting for material coming from distinct domains or categories (see, however, Vergauwe et al., 2009, 2010). The cognitive load effect is therefore barely studied in an interfering context. Our simulations precisely showed that it is likely that this effect exists whatever the level of interference, although it could be modulated by interference (see Plancher et al., in press for similar results observed in older people). Computational modeling is therefore a powerful tool for researchers interested in human cognition because it can test predictions and/or explore research trails before engaging in exhaustive and, thus, costly behavioral testing (Kintsch et al., 1999; Sun, 2008).

The next step would be to assess the model not only on its ability to reproduce the classical effects found in the literature, but also on its ability to simulate the magnitude of these effects. Our goal is to compare model and human data on different experiments, using measures such as the percentage of correct item recall or the serial position curves. Moreover, it is worth noting that similarity-based interference produced by distractors on memoranda is not the sole kind of interference impacting on working memory maintenance. Hence, the present model would encourage future researches to model, for instance, interference within memoranda (benchmark findings A1 to A5, Oberauer et al., 2016). Since memoranda have a distributed representation, it should be possible to reproduce this other kind of interference by implementing a mechanism akin to the one used here to simulate memoranda-distractor interference.

To sum up, TBRS^∗^-I fulfilled the parsimony constraints that have to be considered in a modeling approach, at different levels. First, the implementation was stuck as much as possible to the solid theoretical TBRS model as well as its previous TBRS^∗^ implementation. Second, we propose a similar mechanism for interference and restoration, which is reversible, like their counterpart decay and reactivation. Third, this was achieved using the TBRS^∗^ default parameters and without performing any parameter estimation. TBRS^∗^-I integrates, in a single architecture, two causes of forgetting that were opposed for ages in the literature, while considering a long-term memory component that seems to play a major role in today’s working memory studies (e.g., Loaiza et al., 2014; Rose et al., 2014).

## Author Contributions

All authors listed have made a substantial, direct and intellectual contribution to the work, and approved it for publication.

## Conflict of Interest Statement

The authors declare that the research was conducted in the absence of any commercial or financial relationships that could be construed as a potential conflict of interest.
